# Kooperative Planung von Maßnahmen zur Bewegungsförderung: Neue Wege zur Erweiterung von Handlungsmöglichkeiten – Ergebnisse aus dem Forschungsverbund Capital4Health

**DOI:** 10.1007/s00103-020-03263-z

**Published:** 2020-12-14

**Authors:** Peter Gelius, Hans Peter Brandl-Bredenbeck, Holger Hassel, Julika Loss, Ralf Sygusch, Susanne Tittlbach, Clemens Töpfer, Ulrike Ungerer-Röhrich, Klaus Pfeifer

**Affiliations:** 1 Department für Sportwissenschaft und Sport, Friedrich-Alexander-Universität Erlangen-Nürnberg, Gebbertstr. 123 b, 91058 Erlangen, Deutschland; 2 Institut für Sportwissenschaft, Universität Augsburg, Augsburg, Deutschland; 3 Institut für angewandte Gesundheitswissenschaften, Hochschule Coburg, Coburg, Deutschland; 4 Institut für Epidemiologie und Präventivmedizin, Universität Regensburg, Regensburg, Deutschland; 5 Institut für Sportwissenschaft, Universität Bayreuth, Bayreuth, Deutschland; 6 Institut für Sportwissenschaft, Friedrich-Schiller-Universität Jena, Jena, Deutschland

**Keywords:** Partizipative Gesundheitsförderung, Handlungsmöglichkeiten, Kooperative Planung, Verbundforschung, Bewegungsförderung, Participatory health promotion, Capability approach, Cooperative planning, Consortium research, Physical activity promotion

## Abstract

**Hintergrund:**

Trotz verschiedener nationaler und internationaler politischer Initiativen zur Steigerung des Bewegungsniveaus in der Bevölkerung bleibt die Erarbeitung effektiver Interventionen zur Bewegungsförderung eine Herausforderung. Dabei rücken zunehmend partizipative Ansätze, die zentrale Gruppen und Organisationen in den Settings aktiv in die Erarbeitung konkreter Maßnahmen einbeziehen, in den Fokus der Betrachtung.

**Zielsetzung:**

Dieser Artikel berichtet über die Erfahrungen des Forschungsverbundes Capital4Health mit der Nutzung des partizipativen Ansatzes der „kooperativen Planung“ bei der Entwicklung von Maßnahmen zur Bewegungsförderung für verschiedene Altersgruppen.

**Ergebnisse:**

Der kooperative Planungsansatz wurde von Capital4Health in den Settings Kindertagesstätte, Schule, berufliche Bildung (Automechatronik und Pflege) sowie Kommunen (mit Fokus auf Männer über 50) umgesetzt. Während die zentralen Elemente des Ansatzes in allen Settings umgesetzt wurden, unterschieden sich die Planungsprozesse teils deutlich bezüglich des Spektrums der einbezogenen Gruppen und Organisationen, Anzahl der Teilnehmenden und Sitzungen, der konkret entwickelten Maßnahmen sowie der Evaluationsmethoden und erzielten Wirkungen auf individueller und systemischer Ebene.

**Fazit:**

In der Zusammenschau der bisherigen empirischen Ergebnisse aus den verschiedenen Settings ergibt sich aus Sicht der Projektverantwortlichen in Capital4Health die Schlussfolgerung, dass der Ansatz der kooperativen Planung in sehr verschiedenen Settings funktioniert und erfolgreich gesundheitsförderliche Wirkungen erzeugen kann. Allerdings muss (und kann) er an das Setting angepasst werden, v. a. bei der Einbeziehung von Bevölkerungsgruppen. Die Änderungsbereitschaft von Gruppen und Organisationen ist entscheidend, da Bewegung in den Settings nicht immer oberste Priorität hat. Einzelne Schlüsselfiguren mit hoher intrinsischer Motivation, sich einzubringen, können in diesem Zusammenhang einen entscheidenden Beitrag zum Projekterfolg leisten.

## Abstract (en)

**Background:**

Despite various national and international political initiatives for promoting physical activity at the population level, the development of effective interventions for physical activity promotion remains a challenge. In this context, there is a growing interest in participatory approaches that actively involve central setting actors in the development of specific measures.

**Aim of the article:**

This article reports on the experience made by the Capital4Health research consortium while using a participatory approach called “cooperative planning” to increase capabilities for physical activity across different age groups.

**Results:**

Capital4Health employed the cooperative planning approach in the childcare, school, vocational training (car mechatronics and nursing), and community setting (with a focus on men 50+). While the central elements of the approach were implemented in all settings, planning processes varied significantly with respect to the spectrum of involved actors, the number of participants and sessions, the specific measures developed, the evaluation methods, and the effects achieved at an individual and systems level.

**Conclusion:**

From the perspective of the Capital4Health principal investigators, the preliminary empirical results from the different settings allow for the overall conclusion that the cooperative planning approach can work and generate health promotion impact in very different settings. However, it must (and can) be adapted to the respective setting, especially in relation to involving population groups. Actors’ readiness for change is crucial, as physical activity does not always have top priority in settings. In this context, key individuals can make a decisive contribution to a project’s success.

## Einleitung

Bewegung ist eine zentrale Gesundheitsdeterminante, deren positive Wirkungen u. a. auf Typ-2-Diabetes, bestimmte Krebsarten, kardiorespiratorische Erkrankungen, psychische Beschwerden und andere Beeinträchtigungen gut dokumentiert sind [1–3]. Dennoch bewegen sich große Teile der Bevölkerung zu wenig – so erreicht mehr als die Hälfte der erwachsenen Menschen in Deutschland nicht das national [1] wie international (durch die Weltgesundheitsorganisation WHO) empfohlene Ausmaß an zumindest mäßig anstrengender, aerober körperlicher Aktivität von ca. 2,5 h pro Woche [4, 5]. Globale Trends deuten darauf hin, dass sich dieser Wert in allen Bevölkerungsgruppen künftig noch verschlechtern könnte [5, 6]. Zu wenig Bewegung wird allein in der europäischen Region der WHO jährlich für etwa eine Million Todesfälle verantwortlich gemacht [7]. Dadurch wird Bewegungsmangel zur gesellschaftlichen Herausforderung.

Die Politik hat in den vergangenen Jahren auf verschiedenen Ebenen auf diese Problematik reagiert, u. a. durch Bewegungsempfehlungen [8, 9] und politische Leitlinien auf internationaler Ebene [8–12]. In Deutschland wurde 2016 für die Nationalen Empfehlungen für Bewegung und Bewegungsförderung die international verfügbare Evidenz systematisch gesichtet und an den deutschen Kontext angepasst [1].

Es bleibt jedoch eine Herausforderung, hieraus konkrete Interventionen abzuleiten, die tatsächlich zu nennenswerten Verhaltensänderungen auf Bevölkerungsebene, dauerhaften Strukturveränderungen und zu einer nachhaltigen Verbreitung aktiver Lebensstile führen. Die Wirksamkeit linearer Top-down-Ansätze mit Blick auf diese Zielgrößen wird zunehmend angezweifelt [13]. Stattdessen rücken theoretische und methodische Konzepte in den Fokus, welche das komplexe Zusammenspiel von individuellen und umweltbezogenen Faktoren berücksichtigen. Hierzu zählen z. B. ökologische und biopsychosoziale Modelle [14, 15], Theorien zu Struktur und Handeln [16] oder der Ansatz der Handlungsmöglichkeiten [17, 18]. Zudem mehren sich die Hinweise, dass Ansätze zur Gesundheits- und Bewegungsförderung vor allem dann erfolgreich und nachhaltig sind, wenn die Settingmitglieder, Multiplikator*innen und politische Entscheidungsträger*innen aktiv in die Programmplanung und -implementierung einbezogen werden [19–21]. Basierend auf theoretischen Ansätzen wie interaktivem Wissenstransfer [22, 23], der Co-Produktion von Wissen [24] oder transdisziplinärer Forschung [25] rücken dabei sowohl in der Gesundheitsförderung allgemein [26–28] als auch in der Bewegungsförderung im Besonderen [23, 29, 30] zunehmend partizipative Interventionsformate in den Fokus. Dabei vernetzen sich Fachleute mit Setting-Vertreter*innen und treffen gemeinsam mit ihnen Entscheidungen zu Inhalten und Strategien.

Vor diesem Hintergrund berichtet dieser Artikel über die Erfahrungen, die der Forschungsverbund Capital4Health in den letzten Jahren mit der kooperativen Planung, einem konkreten partizipativen Ansatz zur Entwicklung struktureller Bedingungen und damit einhergehenden neuen Handlungsmöglichkeiten (im Sinne von Amartya Sen, siehe unten) im Bereich Bewegungsförderung gemacht hat [31]. Zudem trägt der Beitrag eine Reihe übergreifender Erkenntnisse zu den Erfolgen, Problemen und Forschungsherausforderungen zusammen, die sich aus Sicht der Projektverantwortlichen aus den bisherigen Projektergebnissen in den einzelnen Settings ergeben. Aus unserer Sicht sind diese Erkenntnisse für all jene von Relevanz, die sich mit der Frage beschäftigen, wie es gelingen kann, mit partizipativen Methoden der Bewegungsförderung auf verschiedenen Ebenen Wirkungen zu erzeugen, d. h. (a) konkrete Maßnahmen, die in verschiedenen Settings passgenau umgesetzt werden können (Outputs), (b) Veränderungen im Bewegungsverhalten bzw. in den Determinanten für Bewegung der Zielgruppe (Outcomes) und (c) systembezogene Veränderungen, die die Handlungsmöglichkeiten für Bewegung erweitern (Impact). Dazu zählen sowohl Forschende als auch politische Entscheidungsträger*innen sowie relevante Gruppen und Organisationen aus verschiedenen Settings der Bewegungsförderung.

## Ansätze und Methoden von Capital4Health

Capital4Health ist ein vom Bundesministerium für Bildung und Forschung (BMBF) finanzierter Forschungsverbund (FKZ01EL1421A), der sich mit der Förderung von aktiven Lebensstilen in unterschiedlichen Settings befasst (Kindergärten, Schulen, Ausbildungsbetrieben, Pflegeheimen, Gemeinden; [32]). Der Verbund wurde ab 2015 zunächst für 3 Jahre gefördert und im Jahr 2018 in einer zweiten Förderphase bis 2021 verlängert. Die theoretische Basis bilden der transdisziplinäre Austausch mit Akteuren aus Politik und Praxis in allen Projektphasen, um so die Passfähigkeit und Nachhaltigkeit seiner Interventionen zu erhöhen [25], sowie das Konzept der „Handlungsmöglichkeiten“ *(Capabilities) *von Amartya Sen [17, 18], welches das Zusammenspiel individueller Kompetenzen, struktureller Einflussfaktoren und dem daraus resultierenden (Bewegungs‑)Verhalten hervorhebt und somit den Blick auf die Wirkungen von Interventionen auf unterschiedlichen Ebenen lenkt.

Die 4 empirischen Teilprojekte von Capital4Health (Tab. 1) etablierten partizipative Gruppenprozesse mit dem Ziel, konkrete Maßnahmen zu planen und umzusetzen, die körperliche Aktivität in dem jeweiligen Setting unterstützen. Als zentrale Methode diente dabei der Ansatz der kooperativen Planung [23, 31]. Er sieht einen dreistufigen Prozess vor, beginnend mit der Identifizierung relevanter Gruppen und Organisationen im Setting und der Zusammenstellung einer Planungsgruppe (Phase 1), gefolgt von der eigentlichen Planungsphase (Phase 2) und der anschließenden Maßnahmenimplementierung (Phase 3). Die Planungsphase (Phase 2) bildet den Kern des Prozesses; sie besteht typischerweise aus einem Brainstorming, der Priorisierung von Zielen, der Entwicklung von spezifischen Maßnahmen und der Verabschiedung eines Maßnahmenkatalogs. Sie umfasst in der Regel zunächst 4–6 Sitzungen der gesamten Planungsgruppe sowie nach Bedarf zusätzliche Treffen von Arbeitsgruppen für einzelne Maßnahmen. Das Konzept legt zudem eine Reihe von Qualitätskriterien fest, z. B. in Form von Regeln für die Moderation, die Kommunikation auf Augenhöhe und gemeinsame Entscheidungsfindung. Der Prozess soll dazu beitragen, dass Interventionen zur Bewegungsförderung passgenau den Settings, den Bedürfnissen der Zielgruppe und den Möglichkeiten der beteiligten Organisationen entsprechen.

**Table 1:** 

Projekt	Setting	Zielgruppe	Zielsetzungen	Quelle
**QueB** („Qualität entwickeln mit und durch Bewegung“)	Kindertagesstätten (Kitas)	Kinder, päd. Fachkräfte	Erhöhung des Bewegungsniveaus von Kindern und päd. Fachkräften	[33]
**Health.edu**	Sekundarschulen	Schüler*innen	Entwicklung der sportbezogenen Gesundheitskompetenz von Schüler*innen	[34]
	Universitäten und Seminarschulen	Angehende Sportlehrkräfte	Entwicklung von Kompetenzen bei Lehrkräften für einen gesundheitsthematischen Sportunterricht	
**PArC-AVE** (“Physical Activity-related Health Competence in Apprenticeship and Vocational Education”)	Berufliche Bildung	Auszubildende	Steigerung der bewegungsbezogenen Gesundheitskompetenz von Auszubildenden	[35]
**ACTION4Men**	Ländliche Kommunen	Männer über 50	Kapazitätsaufbau der Gemeinde zur Steigerung der Motivation für und Partizipation von Männern 50+ an Bewegungsprogrammen	[36]

Die Identifizierung der an der Planung beteiligten Personen erfolgte angepasst an die Bedingungen des jeweiligen Settings, z. B. über bestehende Netzwerke, durch Gespräche mit Verantwortlichen der Einrichtungen oder durch Internetrecherchen und Bedingungsanalysen. Hierdurch wurden (a) Vertreter*innen der im Setting relevanten Zielgruppen, (b) für die Gesundheits- und Bewegungsförderung im Setting relevante Multiplikator*innen, z. B. Expert*innen, Lehrkräfte und Vertreter*innen zentraler Organisationen, sowie (c) politische Entscheidungsträger*innen für den Planungsprozess rekrutiert (Details siehe auch Tab. 2). Die Planungsprozesse wurden durch die wissenschaftlichen Teams der Teilprojekte organisiert und moderiert. Die Forschungseinrichtungen brachten zudem ihr fachliches Wissen in den Prozess ein, während die Setting-Vertreter*innen ihr lokales Wissen sowie ihre Erfahrungen und Netzwerke zur Verfügung stellten.

**Table 2:** 

Projekt	Planungsvorbereitung	Teilnehmende	Anzahl, Zeitraum der Planungstreffen	Prozessschritte
**QueB**	**Landesebene:**	**Landesebene:**	**Landesebene:**	**Landesebene:**
	– Kontaktaufnahme mit der AG Bewegungsförderung – Bestehendes Netzwerk aktivieren/informieren	*Anzahl: n* = 4–8 pro Sitzung	*Sitzungen:* 1–2 pro Jahr *Zeitraum:* 2015–2018	Reflexion bestehender Zertifizierungskonzepte, Begutachtung der Ziele und Maßnahmen der Kitas
	**Regionale Ebene:**	**Regionale Ebene:**	**Regionale Ebene:**	**Regionale Ebene:**
	Informationsveranstaltungen in Coburg und Erlangen	*Anzahl: n* = 3–20 pro Sitzung	Sitzungen: 4 Zeitraum: 2015	Informationen zum QueB-Projekt und -Ansatz
	**Kindertagesstätten (Kitas):**	**Kitas:**	**Kitas:**	**Kitas:**
	Erstgespräche in Kitas	*Anzahl: n* = 2–16 pro Sitzung	*Sitzungen: n* = 6–8 pro Kita *Zeitraum:* 05/2016–07/2017	– Iststandanalyse (Ergebnisse der Kita-Check-App) – Priorisierung von Veränderungsbereichen – Formulierung von SMARTen Zielen und Zielerreichungsskalen – Aufstellung von Maßnahmenplänen mit konkreten Zeitfenstern und Verantwortlichkeiten
**Health.edu**	**Strategische Ebene:**	**Strategische Ebene:**	**Strategische Ebene:**	**Strategische Ebene:**
	– *09/2013:* Kontaktaufnahme mit Bayerischem Staatsministerium für Unterricht und Kultus sowie Ministerialbeauftragten der Realschulen und Gymnasien	*Hintergrund:* Schulleitungen; Sportlehrkräfte; Fachleitungen Sport aller beteiligten Schulen; Seminarlehrkräfte; Dozierende der beteiligten Universitäten; Vertreter*innen des Kultusministeriums und der regionalen Bildungsverwaltung; regionale Fachvertreter*innen; Wissenschaftler*innen	*Sitzungen: n* =2 *Zeitraum:* 07/2015–07/2016	Auftakttreffen bzw. Informationsveranstaltungen
	**Sportunterricht:**	**Sportunterricht:**	**Sportunterricht:**	**Sportunterricht:**
	– *09/2013–10/2014:* Erste Kontaktaufnahme über Sportlehrkräfte und Schulleitungen an weiterführenden Schulen im Raum Bayreuth – *Sommer 2015:* Erneute Kontaktaufnahme nach positivem Förderentscheid, Projektvorstellung bei Schulleitungen/Fachschaften Sport an 4 ausgewählten Schulen (2 Gymnasien, 2 Realschulen) – Syst. Literaturanalyse des sportpäd. Kenntnisstands zum Thema Gesundheit im Sportunterricht	*Vernetzungsgruppe:* *Anzahl: n* = 15 pro Sitzung *Hintergrund: *Beteiligte aller 4 Schulen, u. a. Schulleitungen, Sportlehrkräfte, Fachleitungen Sport, Wissenschaftler*innen, wiss. Hilfskräfte	*Vernetzungsgruppe:* *Sitzungen: n* = 1 *Zeitraum:* 04/2016	– Priorisierung: Verständigung zu (a) sportbezogener Gesundheitskompetenz von Schüler*innen, (b) Zielen, Inhalten, Methoden zum Thema Gesundheit im Sportunterricht – Didaktisch-methodische Maßnahmenplanung, Reflexion der Umsetzung des Themas Gesundheit im Sportunterricht – Planung von Maßnahmen zur Sicherung der Nachhaltigkeit
		*Einzelne Schulen:* *Anzahl: n* = 6–12 pro Sitzung *Hintergrund: *Schulleitung, Sportlehrkräfte, Fachleitung Sport und Schüler*innen, Wissenschaftler*innen, wiss. Hilfskräfte	*Einzelne Schulen:* *Sitzungen: n* = 19 *Zeitraum:* 10/2015–02/2017	
	**Sportlehrkräftebildung:**	**Sportlehrkräftebildung:**	**Sportlehrkräftebildung:**	**Sportlehrkräftebildung:**
	*Sommer 2015:* Kontaktaufnahme mit 2 ausgewählten sportwissenschaftlichen Instituten: Einladung an Dozierende, die in gesundheitsbezogenen Veranstaltungen lehren. Zudem wurden ausgewählte Seminarlehrkräfte für das Fach Sport kontaktiert	*Anzahl: n* = 10–15 pro Sitzung *Hintergrund: *Wissenschaftler*innen, Dozierende, Seminarlehrkräfte Sport, Studiengangskoordinator*in, zentrale Fachleiter*innen, Studierende, Vertretung der Bildungsverwaltung	*Sitzungen: n* = 17 *Zeitraum:* 02/2016–07/2017	– Priorisierung: Verständigung zu (a) sportbezogener Gesundheitskompetenz von Schüler*innen, (b) Zielen, Inhalten und Methoden der Sportlehrkräftebildung – Didaktisch-methodische Maßnahmenplanung – Planung von Maßnahmen zur Sicherung der Nachhaltigkeit
**PArC-AVE**	**Berufsbildungszentrum Pflege (BBZ):**	**BBZ:**	**BBZ:**	**In beiden Settings:**
	– *08/2013:* erste Kontaktaufnahme – *03/2015:* erneute Kontaktaufnahme nach positivem Förderentscheid – *04/2015:* Informationstreffen – *Anschließend:* explorative Situationsanalyse	*Anzahl: n* = 17–22 pro Sitzung *Hintergrund:* Wissenschaftler*innen; stud. Hilfskräfte; Schulleitung; Leitung Pflegeausbildungsprogramm; Leitung Schulfach Krankenpflege; Lehrkräfte; Pflegeschüler*innen; Pflegedirektion der Klinik; Betriebsrat; Behindertenvertretung; Vertretung Krankenkassen	*Sitzungen: n* = 4 *Zeitraum:* 07–10/2016	– Brainstorming von Zielen und Vorschlägen – Kategorisierung von Zielen und Vorschlägen – Priorisierung von Zielen und Vorschlägen – Entwicklung und Definition konkreter Maßnahmen – Festlegung von Maßnahmen, erforderlichen Schritten und Verantwortlichkeiten – Einleitung von Maßnahmen
	**Ausbildungszentrum Kfz-Mechatronik (AZ):**	**AZ:**	**AZ:**	
	– *08/2013:* erste Kontaktaufnahme – *03/2015:* erneute Kontaktaufnahme nach positivem Förderentscheid – *06/2015:* Informationstreffen – *Anschließend:* explorative Situationsanalyse	*Anzahl: n* = 10–14 pro Sitzung *Hintergrund:* Wissenschaftler*innen; stud. Hilfskräfte; Vertretung Arbeitsmedizin; Leitung AZ; Leitung Bereich Automobilausbildung; Vertretung Krankenkrassen; Koordination für Azubi-Projekte; Ausbilder*innen; Auszubildende; Vertretung Jugendausbildung; Betriebsrat; Assistenz der Abteilungsleitung; ext. Dienstleister	*Sitzungen: n* = 4 *Zeitraum:* 07–12/2016	
**ACTION4Men**	**Gemeinde A:**	**Gemeinde A:**	**Gemeinde A:**	**In beiden Gemeinden:**
	– *09/2013:* erste Kontaktaufnahme über Leitung Gesundheitsamt – *06/2015:* erneute Kontaktaufnahme nach positivem Förderentscheid, Projektvorstellung bei Bürgermeister und Gemeinderat – *10/2015:* Informationstreffen in der Gemeinde	*Anzahl: n* = 10–15 *Hintergrund: *Bürgermeister; Vertretung Gemeindeverwaltung; Vertretung Sportvereine; Leitung Gesundheitsamt; weitere Vertreter*innen Gesundheitswesen, Heilberufe, Erwachsenenbildung (VHS); lokale Unternehmen; Männer 50+; Wissenschaftler*innen; stud. Hilfskräfte	*Sitzungen: n* = 9 *Zeitraum:* 10/2015–09/2016	I. Brainstorming von Zielen und Vorschlägen II. Vorstellung Ergebnisse Präerhebungen zu Bedürfnissen, Bedarfen und Barrieren zu Bewegung von Männern 50+ in den Gemeinden III. Kategorisierung von Zielen und Vorschlägen IV. Priorisierung von Zielen und Vorschlägen V. Entwicklung und Definition konkreter Maßnahmen VI. Festlegung der Maßnahmen, der erforderlichen Schritte und Verantwortlichkeiten VII. Einleitung von Maßnahmen VIII. z. T. Evaluation von Maßnahmen
	**Gemeinde B:**	**Gemeinde B:**	**Gemeinde B:**	
	– *11/2015:* erste Kontaktaufnahme über Leitung Gesundheitsamt – *02–03/2016:* Projektvorstellung bei Bürgermeister und Gemeinderat – *04/2016:* Informationstreffen	*Anzahl: n* = 7–14 *Hintergrund: *Bürgermeister; Vertretung Gemeindeverwaltung; Vertretung Sportvereine; Vertretung Landessportverband BLSV; Leitung Gesundheitsamt; weitere Vertreter*innen Gesundheitswesen, Erwachsenenbildung (VHS); Vertretung freiwillige Feuerwehr; freiberufliche Übungsleiterin; Männer 50+; Wissenschaftler*innen; stud. Hilfskräfte	*Sitzungen: n* = 8 *Zeitraum:* 05/2016–02/2018	
	**Gemeindeübergreifend:**	–	–	
	– Quantitative Fragebogenerhebung unter Männern 50+ zu Bedarfen, Bedürfnissen, Barrieren (*N* = 1023) – Semistandardisierte Interviews mit Männern 50+ (*N* = 31) – Literaturreview zu theoretischen Grundlagen erfolgreicher gendersensitiver Bewegungsprogramme für Männer			

Obwohl alle Teilprojekte in Capital4Health demselben Ansatz folgten, war es aufgrund der Besonderheiten der verschiedenen Zielgruppen und Settings erforderlich, die kooperativen Planungsprozesse jeweils teilprojektbezogen anzupassen. Um diese Anpassung systematisch zu erfassen und zu strukturieren, trafen sich die Projektteams in den ersten viereinhalb Jahren des Forschungsverbundes in einer Vielzahl von Feedbackschleifen in verschiedenen ein- oder mehrtägigen Austauschformaten (Projekttreffen, themenbezogene Workshops, jährliche Treffen mit dem internationalen Beirat, transdisziplinäres Steuerungskomitee mit Vertreter*innen aus Politik und Praxis). Dabei wurden die jeweiligen Vorgehensweisen, Erfolgsfaktoren, Herausforderungen der verschiedenen kooperativen Planungsprozesse vorgestellt und diskutiert.

Über die Zeit kristallisierten sich kollektiv übereinstimmende Schlüsselthemen und Erfahrungen mit den Implementierungsprozessen heraus. Diese wurden Ende 2019 im Rahmen mehrerer gesonderter Treffen der Projektkoordinator*innen und mithilfe einer Schablone systematisch zusammengestellt, um daraus die zentralen gemeinsamen Erfahrungen von Capital4Health mit dem Ansatz der kooperativen Planung zu destillieren. Die im Folgenden berichteten Ergebnisse beziehen sich schwerpunktmäßig auf die erste Förderphase von Capital4Health (2015–2018), geben aber auch einen kurzen Ausblick auf die Anpassungen und Veränderungen, die in der zweiten Phase des Verbundes (2018–2021) umgesetzt werden.

## Umsetzung der kooperativen Planung in den Teilprojekten

Tab. 2 gibt einen Überblick über die konkrete methodische Umsetzung des kooperativen Planungsansatzes in den einzelnen Teilprojekten von Capital4Health. Die Planungen fanden in allen Projekten an mehreren Standorten statt, teilweise auch in unterschiedlichen Teilsettings (z. B. Sportunterricht und Sportlehrkräftebildung, Ausbildung im Bereich Pflege und Kfz-Mechatronik) oder in unterschiedlichen Regionen (Kitas im Raum Coburg und Erlangen, verschiedene Gemeinden im Regierungsbezirk Oberpfalz). In 2 Projekten wurden zudem Abstimmungsprozesse auf einer übergeordneten strategischen Ebene durchgeführt (AG Bewegungsförderung im Bereich Kitas, Bayerisches Kultusministerium im Setting Schule und Sportlehrkräftebildung). Alle Teams bereiteten die Planung durch frühzeitige, teilweise mehrfache Kontaktaufnahme mit relevanten Gruppen und Organisationen sowie Informationsveranstaltungen zum Kennenlernen und zur Sensibilisierung für das Thema Bewegung vor, häufig flankiert von einer systematischen Bedingungsanalyse.

Zur Rekrutierung der Teilnehmenden für die Planung setzten die 4 Projekte an die Bedingungen des jeweiligen Settings angepasste Strategien um: Das Spektrum reichte von der Nutzung bestehender Netzwerke über die Kontaktaufnahme mit zentralen politischen Institutionen bis hin zu Informationsveranstaltungen und systematischen Bedingungsanalysen. Zudem wurden eher kleine Planungsgruppen im Schulsetting und in den meisten Kitas eingerichtet. Hingegen wurden im Ausbildungssetting und auf Gemeindeebene sehr große Gruppen etabliert. In allen Projekten ist der Versuch zu erkennen, das gesamte Spektrum der relevanten Gruppen und Organisationen einzubinden, von der Leitungs- über die Arbeitsebene bis hin zu Vertreter*innen der „Zielgruppe“ (mit Ausnahme der Kita-Kinder, da diese als zu jung für die Teilnahme an einem strukturierten Planungsprozess angesehen wurden). Die Anzahl der Treffen variierte ebenfalls stark: Während einige Prozesse (z. B. im Ausbildungssetting) am unteren Ende der vom kooperativen Planungsansatz vorgesehenen Sitzungszahl blieben, gingen andere mit bis zu 10 Sitzungen (Gemeindesetting) deutlich darüber hinaus. Die Prozesse dauerten in der Regel zwischen 6 Monaten und anderthalb Jahren.

Die tabellarische Übersicht zeigt auch, dass alle Prozesse von den Vorgaben des kooperativen Planungsansatzes geleitet waren und die zentralen Schritte umgesetzt wurden. Zugleich finden sich unterschiedliche Schwerpunktsetzungen, z. B. ein Fokus auf die Schaffung eines gemeinsamen Verständnisses zentraler Konzepte zu Beginn der Planung (z. B. Gesundheitskompetenz in Health.edu) oder auf die Nutzung einer bestimmten Erhebungsmethode als Bestandsaufnahme für den Prozess (Kita-Check-App in QueB). Die Abstimmungsprozesse auf der strategischen Ebene folgten in der Regel nicht dem kooperativen Planungsansatz; stattdessen kamen die Beteiligten nur zu einigen weniger stark strukturierten Treffen zusammen.

Ein zentrales Element des kooperativen Planungsansatzes besteht darin, dass Wirkungen nicht primär direkt durch den Planungsprozess erzeugt werden, sondern durch konkrete Maßnahmen zur Gesundheitsförderung, die von der Planungsgruppe erstellt werden und je nach Setting, Teilnehmenden und Prozessverlauf variieren. Die Ergebnisse der Planungsprozesse (Outputs) in den Capital4Health-Teilprojekten sind in Tab. 3 zusammengefasst. Dabei wurde ein breites Spektrum an Maßnahmen generiert, von allgemeinen Organisationsleitlinien (z. B. bewegungsfreundliche Sprache, Anpassung des Organisationsleitbilds) über einmalige Veranstaltungen (Workshops, Informationsveranstaltung) bis hin zu konkreten Bewegungsangeboten, Änderungen am Curriculum und finanzieller Unterstützung der Teilnahme an Bewegungsangeboten. In einigen Fällen griff die Projektleitung steuernd in die Prozesse ein, um die Maßnahmen in eine bestimmte Richtung zu lenken (z. B. durch Vorgaben an die Planungsgruppe, sich auf 3 Maßnahmen pro Kita in QueB oder auf die Erstellung von Unterrichtseinheiten in Health.edu zu beschränken).

**Table 3:** 

Projekt	Output – Bewegungsförderungsmaßnahmen	Outcome – Bewegungsverhalten und Determinanten für Bewegung der Zielgruppe	Impact – systembezogene Veränderungen
**QueB**	*3 Maßnahmen pro Kita, z.* *B.:* – Erweiterung des Materialangebots – Selbstständige Nutzung des Turnraums durch die Kinder – Erweiterung der Bewegungsmöglichkeiten im Außenbereich – Tägliche angeleitete Bewegungsangebote – Qualifizierung der päd. Fachkräfte – Bewegungsfreundliche Sprache – 5‑Minuten-Bewegungseinheiten in jeder Teamsitzung – Einbindung der Eltern, u. a. als „bewegtes Vorbild“	– Zunahme der Schritte pro Stunde bei Kindern und pädagogischen Fachkräften – Weniger Sitzzeiten	– Räume werden geöffnet – Schaffung von mehr Bewegungs- und Differenzierungsmöglichkeiten – Neugestaltung des Außengeländes – Kita-Leiterinnen berichten Veränderungen in der Einstellung und im Verhalten – Päd. Fachkräfte trauen Kindern mehr zu, reflektieren vermehrt eigenes Verhalten und das der Kinder – Mitmachen ist zum Prinzip geworden; Maßnahmen überwiegend fest implementiert (dokumentierte, regelmäßige Rituale)
**Health.edu**	**Sportunterricht:**	**Sportunterricht:**	**Sportunterricht:**
	– Erarbeitung von 10 Sportunterrichtseinheiten zur Förderung der sportbezogenen Gesundheitskompetenz von Schüler*innen an den 4 beteiligten Schulen, davon wurden 9 umgesetzt	– Bewegungsverhalten: nicht erfasst – Sportbezogene Gesundheitskompetenz: Signifikant positive Veränderung; Zunahme der sportbezogenen Gesundheitskompetenz bei Schüler*innen der Interventionsschulen mit kooperativer Planung im Vergleich zu den Kontrollschulen ohne kooperative Planung. Schüler*innen der Interventionsschulen mit erfolgreicher kooperativer Planung profitieren am meisten hinsichtlich der sportbezogenen Gesundheitskompetenz	– Positive Veränderung des didaktischen Handelns und der handlungsleitenden Kognitionen der Sportlehrkräfte (Gesundheitsverständnis, kompetenzorientierte Methoden) – Einbettung des Themas Gesundheit in schulinternen Sportlehrplan (3 Schulen) – Anlegen Materialsammlung der in der Planungsgruppe erarbeiteten methodisch-didaktischen Maßnahmen (2 Schulen) – Vorbereitung des Schulskikurses im Sportunterricht mit Fokus Gesundheit/Fitness (2 Schulen) – Fitnesstag für eine Jahrgangsstufe (1 Schule) – Gesundheitsbezogenes P‑/W-Seminar (1 Schule) keine strukturellen Änderungen (1 Schule)
	**Sportlehrkräftebildung:**	**Sportlehrkräftebildung:**	**Sportlehrkräftebildung:**
	– Erarbeitung von 7 unterschiedlich stark ausdifferenzierten Lehrveranstaltungseinheiten zur Entwicklung von gesundheitsbezogenen Kompetenzen bei Sportlehrkräften (u. a. Tanzen, Schwimmen, Gesundheitsressourcen)	Nicht erfasst	– Positive Veränderung des didaktischen Handelns und der handlungsleitenden Kognitionen der Dozierenden und Seminarlehrkräfte: verstärkter Einsatz kompetenzorientierter Methoden in der Lehre; Gesundheitserziehungsverständnis stärker an Ansprüchen angelehnt – Überarbeitung von Modulen und Lehrveranstaltungen – Informelle und formelle Vernetzung von 1. und 2. Phase der Sportlehrkräftebildung
**PArC-AVE**	**Berufsbildungszentrum Pflege (BBZ):**	**Berufsbildungszentrum Pflege (BBZ):**	**Berufsbildungszentrum Pflege (BBZ):**
	– „Bewegt und Gesund (BUG)“-Stunde: in jeder Klasse wöchent. 90 min in der regulären Schulzeit – Toolbox: PowerPoint-Präsentation mit Übungen für die Gestaltung von Bewegungspausen – Lehrkräftekonferenz: Informationen über das Projekt und die entwickelten Maßnahmen – Weiterqualifizierung von Lehrkräften zu Trainer*innen: Angebot zur Umsetzung der BUG-Stunden – Erhalt/Wiederbesetzung Stelle Sportlehrkraft – Übungsleitungslehrgang für Schüler*innen – Bewegungsförderung als Teil des BBZ-Leitbildes	– Keine substanzielle Verbesserung der bewegungsbezogenen Gesundheitskompetenz – Nicht signifikante Verbesserung der körperlich-sportlichen Aktivität	– BUG-Stunde fest implementiert – Weiterqualifizierung von Lehrkräften zu Trainer*innen umgesetzt – Erhöhter Stellenwert des Themas Bewegung unter den Lehrkräften – Engagierte Leitung und Lehrkräfte, die das Thema Bewegungsförderung vorantreiben
	**Ausbildungszentrum Kfz-Mechatronik (AZ):**	**Ausbildungszentrum Kfz-Mechatronik (AZ):**	**Ausbildungszentrum Kfz-Mechatronik (AZ):**
	– Tutorensystem: Qualifikation von Auszubildenden (Peers) zu Tutor*innen für Bewegungsförderung – Workshop für Ausbilder*innen: Information zum Thema Bewegung und Gesundheit – Ergänzung des vorhandenen „Fit & Gesund“-Workshops um zus. Teil zu Bewegung – Quartalsunterweisung Ausbilder*innen: Informationen zu gesundheitsförderlicher Bewegung – Schaffung von Bewegungsmöglichkeiten am Arbeitsplatz von Auszubildenden – Ergänzung der Betriebsvereinbarung des AZ um das Thema Bewegungsförderung – Anpassung des Leitbildes des AZ	– Keine substanzielle Verbesserung der bewegungsbezogenen Gesundheitskompetenz – Signifikante Verbesserung der körperlich-sportlichen Aktivität über die Zeit, jedoch nicht zwischen Interventions- und Kontrollgruppe	– Tutor*innen-System im dritten Jahr der Umsetzung – „Fit & Gesund“-Workshop für Auszubildende erfolgreich ergänzt – Alle Ausbilder*innen zu gesundheitsförderlicher Bewegung informiert – Erhöhter Stellenwert des Themas Bewegung in der Ausbildung – Engagierte Personen, die das Thema Bewegungsförderung vorantreiben
**ACTION4Men**	**Gemeinde A:**	**Gemeinde A:**	**Gemeinde A:**
	– Neue Website zu Bewegungsangeboten allgemein mit speziellem Fokus auf Männer 50+, zugänglich über Gemeindehomepage – Flyer zu Bewegungskursen für Männer 50+	Keine Aussage zu Veränderungen des Bewegungsverhaltens möglich	– Regelmäßiger Austausch zwischen verschiedenen Interessengruppen, erfolgreiche Akquisition von Ressourcen zur Umsetzung der Maßnahmen – Nur geringfügiger Kapazitätsaufbau
	**Gemeinde B:**	**Gemeinde B**:	**Gemeinde B:**
	– SportCard: subventionierte Nutzung sportvereinsübergreifender Bewegungsangebote für Männer 50+ mit Versicherungsschutz – Schaffung neuer Bewegungsangebote für Männer 50+ durch Vereine, die mit der SportCard oder einzeln genutzt werden können – Informationsveranstaltungen zu (a) Gesundheitswirkungen und motivationspsychologischen Grundlagen regelmäßiger Bewegung und (b) Angeboten der SportCard, inkl. Fitnesstest, Ausprobieren und Bewegungsberatung – Alltagstrainingsprogramm zur Bewegungsförderung für die Zielgruppe 60+	Posterhebung zu Bewegungsverhalten Herbst 2019 durchgeführt (Ergebnisse stehen noch aus)	– Gelungene Zusammenarbeit zwischen verschiedenen Interessengruppen der Gemeinde, erfolgreiche Akquisition von Ressourcen zur Umsetzung der gemeinsam geplanten Maßnahmen, effiziente Arbeitsstrukturen, regelmäßige Erweiterung des Maßnahmenportfolios – Schaffung einer von der Gemeinde finanzierten Stelle (Minijob) zur Koordination und Weiterführung der im Rahmen des Projektes entwickelten Maßnahmen – SportCard als fester Bestandteil der Budget- und Veranstaltungsplanung der Gemeinde – Sehr erfolgreicher Kapazitätsaufbau

*Kita* Kindertagesstätte, *P-/W-Seimnar* Projektseminar/Wissenschaftspropädeutisches Seminar

Die Wirkungen der entwickelten Maßnahmen auf das Bewegungsverhalten der Zielgruppe und seine Determinanten (Outcomes) wurden von den Projekten auf sehr unterschiedliche Weise erhoben. Wie Tab. 3 zeigt, konnten in einigen Settings konkrete Outcomes beobachtet bzw. gemessen werden, z. B. eine Zunahme der Schritte pro Stunde bei Kita-Kindern oder eine erhöhte sportbezogene Gesundheitskompetenz bei Schüler*innen. In anderen Settings sind die Ergebnisse hingegen weniger eindeutig (z. B. bezüglich der körperlich-sportlichen Aktivität von Auszubildenden) oder es konnten keine Wirkungen gemessen werden (bewegungsbezogene Gesundheitskompetenz von Auszubildenden).

Auf der Systemebene konnten die Projekte in verschiedenen Bereichen positive Veränderungen der strukturellen Bedingungen und der damit einhergehenden neuen Handlungsmöglichkeiten für Bewegung (Impact) nachweisen, u. a. bezüglich der dauerhaften Verankerung von Maßnahmen in Organisationsprozesse und -strukturen (z. B. Lehrpläne, Leitbilder, Tutorensysteme), der Einstellung und des Verhaltens relevanter Multiplikator*innen (z. B. Lehrkräfte und Leitungspersonal, Etablierung von „Kümmer*innen“ für das Thema Bewegung, Kooperation zwischen relevanten Gruppen und Organisationen) und des Bewegungsverhaltens der Multiplikator*innen selbst (Kita-Personal). Diese Ergebnisse können zum Teil auf die konkret umgesetzten Maßnahmen (Output) und zum Teil auf die Interaktion im kooperativen Planungsprozess an sich zurückgeführt werden.

## Zentrale Erkenntnisse aus Capital4Health

Detailergebnisse zu den Planungsprozessen in den verschiedenen Settings von Capital4Health und zu ihrer Wirkung im Sinne von Outputs, Outcomes und Impact sind verschiedentlich an anderer Stelle ausführlich dargelegt worden [33–38]. Im Folgenden sollen einige übergreifende Erkenntnisse berichtet werden, die sich aus Sicht der Projektverantwortlichen (Principal Investigators, kurz PIs) aus der Zusammenschau dieser Einzelergebnisse ergeben und über den Verbund hinaus für künftige Aktivitäten in der partizipativen Gesundheits- und Bewegungsförderung relevant sind. Sie wurden von den PIs in einem mehrmonatigen Austauschprozess in Form von persönlichen Gesprächen, gemeinsamen Sitzungen und der Arbeit an systematischen Übersichtsdokumenten zusammengetragen.

### Kooperative Planung funktioniert in verschiedenen Settings, aber sie muss (und kann!) angepasst werden

Die Erfahrung aus Capital4Health zeigt, dass der Ansatz der kooperativen Planung in verschiedenen Settings und vor dem Hintergrund verschiedener wissenschaftlicher (Teil‑)Disziplinen einsetzbar ist. Dabei können Strukturen und Kompetenzen für Bewegung verbessert werden. Der Ansatz ist allerdings gerade in der Anbahnungsphase zeit- und ressourcenintensiv. Zudem sind teilweise weitreichende Anpassungen an die Bedingungen des jeweiligen Settings notwendig, z. B. bezüglich der angesteuerten Ebenen, der Gruppengröße und Anzahl der Treffen, der Schwerpunktsetzung und der Steuerung durch die Projektleitung. Ebenso unterscheiden sich die in den Planungsprozessen entwickelten Maßnahmen zwischen den Settings deutlich. Umgekehrt unterstreicht dies jedoch auch, dass Bewegungsförderung in unterschiedlichen Lebenswelten nicht durch standardisierte „Einheitsinterventionen“ gelingen kann und dass stattdessen flexible Ansätze und Methoden nötig sind. Aus Capital4Health ist ersichtlich geworden, dass die kooperative Planung ein Ansatz ist, der einerseits Anpassungen erlaubt und andererseits Projekten in verschiedenen Settings einen gemeinsamen methodischen Rahmen bietet und eine enge wissenschaftliche Kooperation ermöglichen kann.

### Bewegung hat nicht notwendigerweise Priorität in den ausgewählten Settings

Die Evidenz für den gesundheitlichen Nutzen von Bewegung steht außer Frage. Dennoch lehrt die Erfahrung von Capital4Health, dass Vertreter*innen verschiedener Settings das Thema „aktiver Lebensstil“ zwar als generell interessant, jedoch nicht immer als relevant für ihren Kontext und in ihrer persönlichen Verantwortung liegend wahrnehmen. Während beispielsweise in Kitas tendenziell große Einigkeit über die Wichtigkeit der Steigerung der Bewegungszeiten für die Kinder herrscht, wird auf kommunaler Ebene oft auf das bereits bestehende, umfassende Angebot der örtlichen Sportvereine verwiesen. In anderen Settings besteht auch Einigkeit zwischen den Beteiligten darüber, dass die Steigerung der Bewegungszeit nicht die primäre Zielrichtung darstellt, z. B. in den Bereichen Sportunterricht und berufliche Bildung, wo Schüler*innen in erster Linie gesundheitsbezogene Kompetenzen für die körperliche Aktivität und Sport erwerben sollen.

Insgesamt erscheint es wichtig, Gruppen und Organisationen in allen Lebenswelten für die verschiedenen Aspekte von Bewegung zu sensibilisieren und Kapazitäten für den Umgang mit diesem komplexen Thema zu schaffen. Zugleich ist es nötig, dass die Wissenschaft auf die Sichtweise der Beteiligten eingeht und versucht, die ggf. unterschiedlichen Anliegen der jeweiligen Planungsgruppen mit jenen der Forschung zu verbinden. Zudem ist bereits in der Designphase von Projekten zu klären, ob Bewegung als zentrale Outcome-Variable definiert werden soll, und die Interventionen müssen unter Nutzung flexibler theoretischer Ansätze (z. B. dem Capability-Ansatz) konzeptualisiert werden.

### Die Änderungsbereitschaft des jeweiligen Settings ist entscheidend

Kooperative Planungsgruppen sollen Entscheidungen treffen und Maßnahmen im jeweiligen Setting umsetzen, was notwendigerweise Änderungen in den Strukturen zur Folge hat. Der zielgerichtete Charakter des kooperativen Planungsprozesses sowie die Qualitätskriterien (z. B. die „Spielregeln“ für die Kommunikation zwischen den Beteiligten) können einen Beitrag dazu leisten, dass nicht nur das Endergebnis positiv ist, sondern auch die Interventionsplanung selbst von den involvierten Gruppen und Organisationen positiv wahrgenommen wird [31].

Dennoch ist aus der Literatur bekannt, dass Settings nicht immer zu Veränderungen bereit sind und dass die Änderungsbereitschaft („organisational readiness for change“ [39]) eine große Rolle für den Erfolg von Maßnahmenplanungen spielt. Diese Erfahrung wurde durchgängig in allen Teilprojekten von Capital4Health gemacht. So wirkte beispielsweise die Mehrzahl der Kitas engagiert in den kooperativen Planungsprozessen mit und ermöglichte so z. B. längere Bewegungszeiten drinnen wie draußen und eine bewegungsfreundliche Umgestaltung der Räumlichkeiten. In einigen Kitas reflektierten die pädagogischen Fachkräfte darüber hinaus auch über ihr eigenes Handeln und brachten Veränderungen auf der eigenen Verhaltensebene auf den Weg. Umgekehrt führten Personalmangel und Schwierigkeiten im Team oder auf der Leitungsebene dazu, dass andere Kitas überhaupt nicht an der kooperativen Planung teilnahmen. Im Setting Sportlehrkräftebildung zeigte sich teilweise eine gewisse „Sättigung“ bezüglich der Gesundheitsthematik und eine entsprechend geringe Änderungsbereitschaft. In einer der beteiligten Gemeinden konnten die Teilnehmenden der Planungsgruppe keinen wesentlichen Bedarf zur Strukturänderung feststellen. Sie waren daher mit ersten, relativ einfachen Maßnahmen (z. B. Erstellung eines Info-Flyers) zufrieden und verzichteten auf die Realisierung weitergehender Interventionen. Settingübergreifend kritisierten die Teilnehmenden den mitunter zeitintensiven Planungsprozess sowie den vergleichsweise späten Übergang von der allgemeinen Vorbereitung hin zur Entwicklung konkreter Maßnahmen.

Insgesamt erscheint es für künftige Projekte sinnvoll, die Veränderungsbereitschaft des jeweiligen Settings so früh wie möglich konkret zu prüfen, z. B. über qualitative Interviews oder die Nutzung von Messinstrumenten für Team oder Community Readiness [40, 41]. Anhand dieser Informationen ist zu entscheiden, ob sich Projekte mit kooperativen Planungsprozessen auf entsprechend vorbereitete Umwelten beschränken sollen oder ob in nicht bereiten Settings zunächst andere, vorbereitende Interventionen (z. B. Teambuilding, Awareness Raising (Bewusstseinsbildung)) durchgeführt werden.

### Die Einbeziehung von Zielgruppen erfordert angepasste Vorgehensweisen

Ziel von Capital4Health war es u. a., in den kooperativen Planungsgruppen die partizipative Interaktion zwischen Wissenschaftler*innen, Multiplikator*innen und politischen Entscheidungsträger*innen in den Settings sowie der eigentlichen Zielgruppe zu ermöglichen, und zwar mit dem Ziel der konkreten Maßnahmenplanung. Damit geht der Ansatz potenziell über andere partizipative Vorgehensweisen hinaus, die sich entweder auf frühere Phasen eines Entwicklungsprozesses beziehen (z. B. bei der Datensammlung [42]) oder die auf Arnsteins „Partizipationsleiter“ nur niedrigere Stufen erzielen (z. B. Information). Die kooperative Planung kann demgegenüber höher eingeordnet werden (Stufe 6, „Mitbestimmung“; Stufe 7, „teilweise Entscheidungskompetenz“ oder gar Stufe 8, „Entscheidungsmacht“ [43, 44]).

Unsere Erfahrungen zeigen jedoch, dass die Einbeziehung von Vertreter*innen der Zielgruppen in einigen Settings nur schwer möglich ist und je nach Umfeld in unterschiedlichen Formaten umgesetzt werden muss. So wurde eine Teilnahme von Kindern an den kooperativen Planungssitzungen in den Kitas von vorne herein nicht in Betracht gezogen. In den Schulen waren Schüler*innen an den meisten Planungsformaten beteiligt; zusätzlich wurde versucht, der Zielgruppe systematisch mehr Gehör zu verschaffen, z. B. über die Einbeziehung der Schülermitverantwortung (SMV). In den Betrieben nahmen an den kooperativen Planungsgruppen mit Ausbildungsleitern und Betriebsärztin auch Auszubildende teil, für die es jedoch nicht einfach war, ihre Meinung offen im Beisein der aktuellen und künftigen Vorgesetzten zu äußern.

Am schwierigsten erwies sich die Einbeziehung der Zielgruppe „Männer 50-plus“ in den Gemeinden. Hier gelang es zwar, Männer dieser Altersgruppe in die kooperative Planungsgruppe zu integrieren, die bereits Funktionen in der Gemeinde oder in örtlichen Vereinen innehatten; eine dauerhafte Einbeziehung von Männern „ohne Funktion“ in die Planungsgruppe oder ein eigenes Versammlungsformat konnten jedoch nicht realisiert werden.

Künftige Projekte sollten beachten, dass sich zielgerichtete Entwicklungsansätze wie die kooperative Planung nicht in allen Settings für eine umfassende Einbindung der Zielgruppe eignen und dass ggf. eine Anpassung des Vorgehens nötig ist, um eine gewinnbringende Partizipation zu ermöglichen. Zudem ist zu bedenken, dass die Partizipation von Zielgruppen aufgrund der Komplexität des Prozesses immer nur über wenige ausgewählte Vertreter*innen stattfinden kann. Andere Ansätze (z. B. Town Hall Meetings) ermöglichen breitere Partizipation, sind aber für die Entwicklung konkreter Maßnahmen u. U. weniger geeignet [45].

Ein weiterer kritischer Punkt ergibt sich aus der aktuellen Projektförderlogik: Für die Einbeziehung relevanter Gruppen und Organisationen schon in der Phase der Antragstellung stehen in der Regel keine Fördermittel zur Verfügung; die Projektanträge sind daher meist nicht partizipativ entwickelt und folgen einer vorwiegend wissenschaftlichen Logik, welche die Erzielung nachhaltiger Wirkungen im Setting unter Umständen einschränkt [46].

### Einzelne Schlüsselfiguren können entscheidend zum Projekterfolg beitragen

Die Aktivität und Effektivität von kooperativen Planungsgruppen hängen in vielen Fällen von einzelnen Personen ab, die ein intrinsisches Interesse an körperlicher Aktivität haben und bereit sind, einen hohen Einsatz für die Umsetzung entsprechender Maßnahmen zu erbringen. Obwohl Personen in bestimmten Funktionen theoretisch als prädestiniert für diese Rolle erscheinen, kristallisierten sich die tatsächlichen „Kümmer*innen“ oder „Champions“ [47, 48] in Capital4Health erst durch ihr persönliches Agieren im Planungsprozess heraus oder wuchsen sogar (siehe oben) erst im Verlauf der Projekte in diese Rolle hinein.

So erwies sich beispielsweise eine Betriebsärztin als wesentliche „treibende Kraft“ für den Fortgang der kooperativen Planung im Setting berufliche Bildung. In einer der beteiligten Gemeinden übernahm der Bürgermeister die Führung der Planungsgruppe und ermöglichte die effiziente Umsetzung vieler Maßnahmenideen, während sein Amtskollege in der anderen Gemeinde zurückhaltend war und den Planungsfortgang durch Bedenken teilweise eher verlangsamte. Die möglichst frühe Identifikation derartiger „Champions“ und ihre Unterstützung durch die beteiligten Wissenschaftler*innen erscheint als entscheidend für den Erfolg von Planungsgruppen und stellt eine zentrale Aufgabe für künftige kooperative Projekte der Bewegungsförderung dar.

## Ausblick

Die Weiterfinanzierung von Capital4Health für eine zweite Projektphase erlaubt den Teilprojekten eine Fortsetzung ihrer Arbeit bis 2021. Dabei verfolgen sie vorrangig das Ziel, ihre erfolgreichen Interventionen auf andere Standorte, zusätzliche Settings oder auch auf „höhere“ politische Ebenen (z. B. Regierungsbezirk, Bundesland, Dachorganisationen) zu übertragen, wobei sie weiterhin den Ansatz der kooperativen Planung nutzen. Das Teilprojekt QueB setzt einerseits auf eine stärkere regionale Vernetzung von Kitas, Trägern und Erzieher*innen-Ausbildung und andererseits auf eine Kooperation mit der „Plattform Ernährung und Bewegung“ (peb) zur bundesweiten Disseminierung. In Health.edu wird die Intervention an ausgewählten Sekundarschulen (sogenannten Modellschulen) weitergeführt. Zudem wird die kooperative Planung auf neue Sekundarschulen ausgeweitet und ein weiterer Schultyp (Grundschule) kommt hinzu. PArC-AVE (Tab. 1) weitet seine Intervention auf eine andere Abteilung des Industrieunternehmens aus Phase 1 aus und kooperiert mit zusätzlichen staatlichen Pflegeschulen und dem zuständigen Kultusministerium. Action4Men konzentriert sich in Phase 2 auf eine seiner beiden Modellkommunen, hat aber eine weitere Gemeinde angeworben und kooperiert zudem auf höherer Ebene mit der Bezirksregierung der Oberpfalz bzw. Dachorganisationen des Sports. Ziel ist eine Skalierung („scaling up“ [49]) und Verstetigung von kooperativ geplanten Interventionen über die zeitliche Begrenzung von einzelnen Pilotprojekten hinaus.

Neben individuellen Anpassungen in den verschiedenen Projekten stellen sich auch gemeinsame Herausforderungen für den Forschungsverbund als Ganzes. Dazu gehören Wege zu einer verbesserten Einbeziehung von Bevölkerungsgruppen in die Planungsprozesse der jeweiligen Settings – z. B. durch Kombination der kooperativen Planung mit Ansätzen aus dem Bereich „Citizen-Science“ [42] –, eine verstärkte Interaktion zwischen den einzelnen Teilprojekten (z. B. bei der Messung von Projektergebnissen) sowie die gemeinsame Weiterentwicklung der theoretischen und methodischen Arbeit. Letzteres betrifft sowohl die Operationalisierung und Messung des Ansatzes der „Handlungsmöglichkeiten“ als auch die Methode der kooperativen Planung. Idealerweise können die Erfahrungen von Capital4Health hier zu einer Überarbeitung bzw. Erweiterung des Ansatzes beitragen, die einen noch besseren Austausch zwischen Zielgruppen, Praxis, Politik und Wissenschaft in unterschiedlichen Settings erlaubt und die Effektivität von lebensweltnahen Interventionen zur Bewegungsförderung dadurch weiter erhöht.
